# Association of Social Capital With Tuberculosis: A Community-Based Cross-Sectional Analytical Study in South India

**DOI:** 10.7759/cureus.46660

**Published:** 2023-10-07

**Authors:** Premkumar Ramasubramani, Sitanshu Sekhar Kar, Sonali Sarkar

**Affiliations:** 1 Department of Preventive & Social Medicine, Jawaharlal Institute of Postgraduate Medical Education & Research, Puducherry, IND

**Keywords:** social determinant of health, health, disease, tuberculosis, social infrastructures, support system, social integration, social capital

## Abstract

Background: Social capital denotes the relationships, networks, norms and values in the community. A high level of social capital positively improves health through a supportive social system. Illnesses affect health and social relationships. One such disease is tuberculosis (TB), known for its social stigma. India has the highest burden of morbidity and mortality due to TB. The assessment of social capital would highlight the importance of a supportive environment in reducing the disease burden and bringing better treatment outcomes.

Methods: A cross-sectional exploratory analytical study was conducted in two primary health centers in Puducherry between February 2020 and March 2021. Considering the feasibility and resource constraints, we assessed the social capital between 50 newly diagnosed pulmonary tuberculosis (PTB) patients, their age- and gender-matched 50 household contacts (HHCs) and 50 PTB patients who completed treatment a year before. The HHC was either the marital partner or sibling of the newly diagnosed PTB patients selected for comparison as their exposure to infection would be similar to those diseased but did not develop the illness. Social capital and its domains were assessed using the World Bank's social capital questionnaire. Sociodemographic characteristics and social capital domains were compared using a chi-squared test. Mean standardized Z-scores of the domains were compared using one-way analysis of variance (ANOVA). A p-value of <0.05 is taken as significant.

Results: Most participants from each group belonged to lower socioeconomic strata and were males (80%). The overall level of social capital was low among the newly diagnosed PTB patients, especially the group and network and trust and solidarity domains. The mean standardized Z-scores of social capital were the highest among the HHCs, followed by the treatment-completed PTB patients. There was no consistent pattern, but the trust and solidarity domain showed a statistically significant difference.

Conclusion: A low level of social capital and its domains were seen among the newly diagnosed PTB patients. However, better scores among the HHCs and the treatment-completed patients infer a negative association between social capital and TB. Thus, higher social capital preserves and improves health. Therefore, caregivers and disease-cured patients can be utilized as a social support system for current diseased patients and improve their health status.

## Introduction

Contributing to over 1.3 million deaths globally, tuberculosis (TB) ranked as the leading cause of deaths in 2020 and one of the top 10 causes of deaths [1]. India accounted for 26% of the global TB burden with estimated incident cases of TB in India being 26 lakhs and 4.4 lakh deaths in the year 2020 [2].

Along with determinants, such as behavioral factors, biological factors and socioeconomic status, social determinants contribute to the physical and mental well-being of individuals. Disease onset, progression and death depend on the social determinants of health. The concept of going beyond the individual determinants of health, which influence the lives of people, is known as social capital [3].

Social capital refers to the social norms, relationships, networks and values that affect the functioning and development of the society [4]. The social capital of a TB patient will also be measured, which can help us quantify the social support received by them [5]. It defines how strongly involved an individual is in his community in terms of trust, solidarity, social cohesion, networking and decision-making. A good social capital indicates a good interaction of the individual with the society, which is established as a beneficial factor for their health [6]. A disease like TB has a severe social impact at the individual level and family level, leading to impoverishment.

In a country like India, where people from various religions, castes and political ideologies co-exist, studying the social capital of individuals will bring to light the importance of good support within the community. Low social capital, in terms of cohesion and network, was found to be associated with all forms of mortality, and social participation represents a strong domain of social capital [7].

Getting to know the social capital and its domains will help in identifying deficiencies in the social environment of the study participants. Improving social capital would improve the well-being and survival of TB patients. The findings of the study will highlight the importance of the social environment that could greatly reduce the disease burden and improve the treatment outcome. This study aimed to compare the social capital among newly diagnosed pulmonary tuberculosis (PTB) patients, their household contacts (HHCs) and PTB patients who completed treatments for more than a year.

## Materials and methods

We conducted an exploratory cross-sectional analytical study between March 2020 and February 2021 in two high-TB-burden primary health centres (PHCs) in the union territory of Puducherry, India. Under the National Tuberculosis Elimination Programme, diagnosis and treatment were provided free of cost. Persons diagnosed with TB at the designated microscopy centre were referred to their nearby PHC for initiation of treatment after screening for comorbidities, such as type II diabetes and HIV. The sociodemographic details, clinical course, medication adherence and comorbidity profile were maintained at the PHC and followed up using treatment cards.

There were three groups in this study: group 1 (newly diagnosed adult PTB patients), group 2 (HHCs of the newly diagnosed adult PTB patients) and group 3 (PTB patients who had completed treatment one year before the commencement of the study). Participants in group 1 were recruited within four weeks of the initiation of treatment. We enrolled participants with the above inclusion criteria. If either newly diagnosed adult PTB patients or their respective HHC did not express willingness, the other was not recruited. This method was used to maintain the link between groups 1 and 2.

The study was initiated after receiving approval from the Institutional Ethical Committee of Jawaharlal Institute of Postgraduate Medical Education and Research (approval no. JIP/IEC/2019/240). In addition, the State TB Operational Research Committee under the programme approved the study (approval no. PSHM/RNTCP/Acc/S2/2019-20/854). Written consent was obtained from all participants enrolled in the study. We incorporated consecutive sampling for recruitment. Data were collected regarding the sociodemographic variables, such as age, gender, education, occupation and marital status, and to assess the social capital, we used the World Bank's social capital questionnaire [8]. The social capital questionnaire was culturally adapted, translated to Tamil and pilot-tested before data collection. There were 27 core questions for the measurement of social capital in the World Bank questionnaire, which covers six domains, namely, groups and networks, trust and solidarity, collective action and cooperation, information and communication, social cohesion and inclusion and empowerment and political action [8].

We calculated the sample size for the primary study where, considering the feasibility and resource constraints, 50 newly diagnosed PTB patients were recruited into group 1 and 100 healthy age- and gender-matched individuals (50 HHCs and 50 previously treated PTB patients) were recruited into groups 2 and 3 [9].

Data were collected in EpiCollect5 (developed by Imperial College, London, and funded by Wellcome Trust) and analyzed using STATA v14 (StataCorp, TX, USA, 2015, Stata Statistical Software, release 14). The sociodemographic characteristics were expressed as frequencies and percentages. The social capital scores were expressed as quartiles. A chi-squared test was used to compare the parameters between groups. The scores were also standardized using Z-score transformation and presented as the mean (standard error) of Z-scores for comparison between groups. One-way analysis of variance (ANOVA) was used to compare the means of the Z-scores. A p-value of <0.05 was considered to be significant.

## Results

We contacted 67 newly diagnosed PTB patients and their HHC and 96 treatment-completed PTB patients to enroll 50 participants in each group. The response rate was 74.6% among the newly diagnosed PTB patients and their HHCs and 52.1% among the treatment-completed PTB patients.

The mean (standard deviation (SD)) age (years) of the newly diagnosed PTB patients, their HHCs and treatment-completed PTB patients were 38.4±6.8, 36.6±5.3 and 38.9±7.1, respectively. Almost three-fourths of the participants were less than 45 years of age. Most of the study participants were males (80%) and were literate. More than 35% of the participants had a graduation/post-graduation degree. The majority belonged to the lower-middle and upper-middle socioeconomic classes as measured by the modified Kuppusamy scale [10]. Over 60% of the participants belonged to nuclear families and more than three-fourths of them were married (Table 1).

**Table 1:** Sociodemographic characteristics of the study participants, N=150 Group 1: newly diagnosed tuberculosis patients; group 2: household contacts of newly diagnosed tuberculosis patients; group 3: treatment-completed tuberculosis patients * modified Kuppusamy scale, 2021. † chi-squared test

Characteristics	Group 1 (n=50) n (%)	Group 2 (n=50) n (%)	Group 3 (n=50) n (%)	P-value^†^
Age categories (years)				
<35 years	20 (40)	12 (24)	15 (30)	0.22
>=35 years	30 (60)	38 (76)	35 (70)	
Gender				
Male	40 (80)	40 (80)	40 (80)	1.00
Female	10 (20)	10 (20)	10 (20)	
Educational status				
No formal schooling	4 (8)	2 (4)	6 (12)	0.68
Primary (1-4)	6 (12)	7 (14)	9 (18)	
Secondary (5-10)	12 (24)	13 (26)	12 (24)	
Higher secondary (11-12)	6 (12)	11 (22)	6 (12)	
Graduate/post-graduate	22 (44)	17 (34)	17 (34)	
Socioeconomic status*				
Upper middle (II) (16-25)	9 (18)	9 (18)	6 (12)	0.44
Lower middle (III) (11-15)	26 (52)	26 (52)	36 (72)	
Upper lower (IV) (5-10)	14 (28)	14 (28)	7 (14)	
Lower (V) (<5)	1 (2)	1 (2)	1 (2)	
Type of family				
Nuclear	32 (64)	32 (64)	28 (56)	0.91
Joint	13 (26)	13 (26)	15 (30)	
Three generations	5 (10)	5 (10)	7 (14)	
Marital status				
Unmarried	9 (18)	7 (14)	6 (12)	0.78
Married	40 (80)	43 (86)	43 (86)	
Widow/widower/divorced/separated	1 (2)	0 (0)	1 (2)	

Table 2 shows the quartile-wise distribution of the social capital and its domains among the study groups. A higher proportion of the newly diagnosed TB patients was present in the poorest quartiles of social capital and six domains. In the group and network domain, 19 newly diagnosed TB patients were in the lowest quartile, which was nearly double that of the richest quartile (38% vs. 18%). HHCs and treatment-completed PTB patients were roughly equally distributed in the quartiles. For the trust and solidarity domain, about 36% of the newly diagnosed TB patients were in the poorest quartile, but a higher proportion was in the third quartile among the HHCs and treatment-completed PTB patients. The highest proportion of the participants was in the poorest quartile in all the groups for the collective action and cooperation domain (44% vs. 38% vs. 38%) and empowerment and political action domain (48% vs. 36% vs. 38%). Similarly, in the information and communication domain, there was roughly an equal distribution in the poorest quartile (30% vs. 36% vs. 36%) and third quartile (38% vs. 36% vs. 36%) among all the three groups. In the social cohesion domain, a roughly equal distribution was noticed in all quartiles among all the three groups. For the overall social capital, a relatively higher proportion of newly diagnosed TB patients (32%) were in the poorest quartile, followed by treatment-completed TB patients (26%) and HHCs of newly diagnosed TB patients (20%).

**Table 2:** Distribution of social capital and its domains among the study participants; N=150 Group 1: newly diagnosed tuberculosis patients; group 2: household contacts of newly diagnosed tuberculosis patients; group 3: treatment-completed tuberculosis patients * chi-squared test

Domains	Group 1 (n=50) n (%)	Group 2 (n=50) n (%)	Group 3 (n=50) n (%)	P value*
Groups and networks				
1^st^ (poorest)	19 (38)	13 (26)	12 (24)	0.637
2^nd^	9 (18)	14 (28)	12 (24)	
3^rd^	13 (26)	11 (22)	12 (24)	
4^th^ (richest)	9 (18)	12 (24)	14 (28)	
Trust and solidarity				
1^st^ (poorest)	18 (36)	11 (22)	15 (30)	0.002
2^nd^	14 (28)	8 (16)	11 (22)	
3^rd^	9 (18)	29 (58)	22 (44)	
4^th^ (richest)	9 (18)	2 (4)	2 (4)	
Collective action and cooperation				
1^st^ (poorest)	22 (44)	19 (38)	19 (38)	0.942
2^nd^	5 (10)	9 (18)	9 (18)	
3^rd^	10 (20)	10 (20)	10 (20)	
4^th^ (richest)	13 (26)	12 (24)	12 (24)	
Information and communication				
1^st^ (poorest)	15 (30)	18 (36)	18 (36)	0.997
2^nd^	9 (18)	8 (16)	8 (16)	
3^rd^	19 (38)	18 (36)	18 (36)	
4^th^ (richest)	7 (14)	6 (12)	6 (12)	
Social Cohesion				
1^st^ (poorest)	14 (28)	13 (26)	12 (24)	0.979
2^nd^	16 (32)	14 (28)	15 (30)	
3^rd^	10 (20)	9 (18)	11 (22)	
4^th^ (richest)	10 (20)	14 (28)	12(24)	
Empowerment and political action				
1^st^ (poorest)	24 (48)	18 (36)	19 (38)	0.809
2^nd^	6 (12)	9 (18)	10 (20)	
3^rd^	7 (14)	11 (22)	9 (18)	
4^th^ (richest)	13 (26)	12 (24)	12 (24)	
Social Capital (Total score)				
1^st^ (poorest)	16 (32)	10 (20)	13 (26)	0.901
2^nd^	11 (22)	14 (28)	12 (24)	
3^rd^	11 (22)	14 (28)	12 (24)	
4^th^ (richest)	12 (24)	12 (24)	13 (26)	

**Figure 1:**
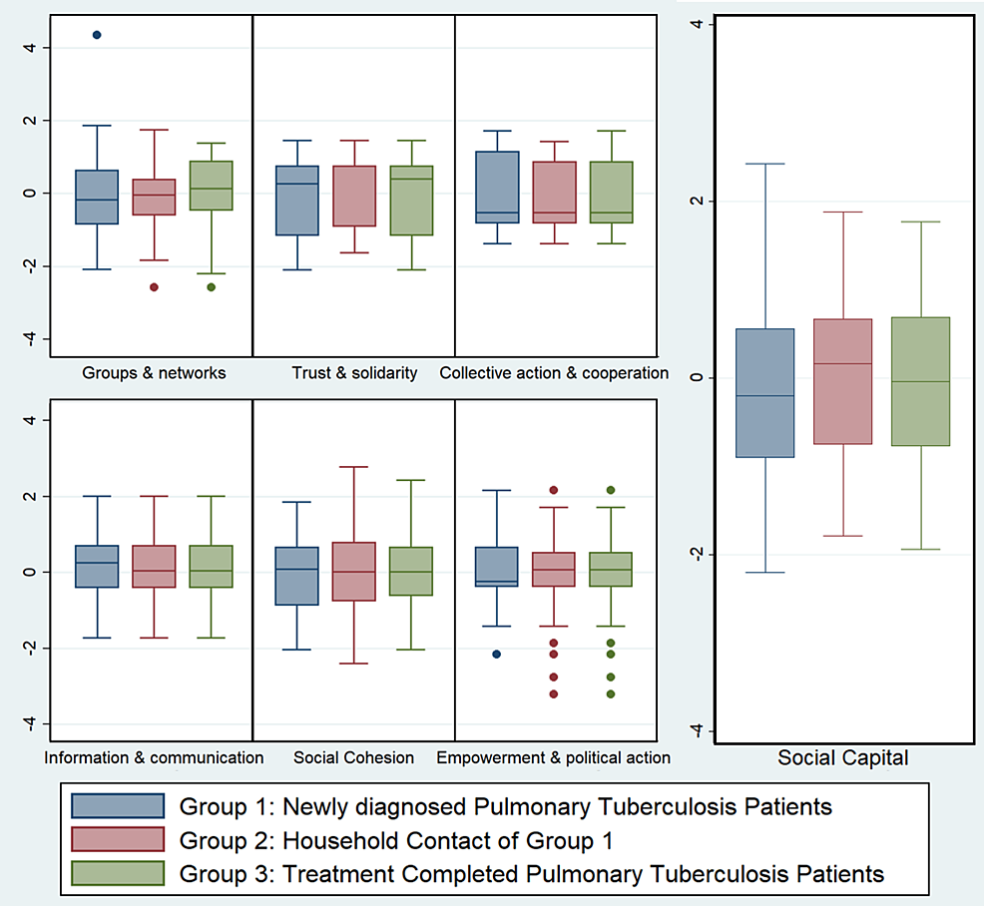
Box-whisker plot of the Z-score distribution of social capital and its domains among the study participants; N=150 The Y-axis denotes the z-scores of the social capital levels and its domains.

The mean standardized Z-score of the social capital was the highest among the HHCs, followed by the treatment-completed PTB patients and the newly diagnosed PTB patients (Table 3). A similar kind of trend was seen in the trust and solidarity domain (-0.13 vs. 0.18 vs. -0.05). Treatment-completed PTB patients had the highest score in the group and network (-0.05 vs. -0.01 vs. 0.06) and social cohesion domains (-0.09 vs. 0.04 vs. 0.05) compared to the HHCs and newly diagnosed PTB patients. The respective scores for the collective action and cooperation domain were 0.01, -0.01 and 0.03; for information and communication were 0.07, -0.04 and -0.04; and for empowerment and political action were 0.01, 0.02 and -0.03. The maximum difference in the Z-scores was seen between the newly diagnosed PTB patients and their HHCs in the trust and solidarity (-0.19) and social cohesion domains (-0.13) in the negative direction, i.e. newly diagnosed PTB patients had a worse score than HHCs, and the information and communication domain (0.11) in the positive direction, i.e. newly diagnosed PTB patients had a better score than HHCs. Between the newly diagnosed PTB patients and the treatment-completed PTB patients, the maximum difference in the Z-scores was in the social cohesion (-0.14) and group and network domains (-0.11) in the negative direction, i.e. newly diagnosed PTB patients had a worse score than treatment-completed PTB patients, and the information and communication domain (0.13) in the positive direction, i.e. newly diagnosed PTB patients had a better score than the treatment-completed PTB patients. The pictorial representation of the mean standardized Z-score of the social capital and its domains is provided in Figure 1.

**Table 3:** Mean values of social capital and its domains among the study participants; N=150 Group 1: newly diagnosed tuberculosis patients; group 2: household contacts of newly diagnosed tuberculosis patients; group 3: treatment-completed tuberculosis patients * one-way analysis of variance (ANOVA)

Domains	Z-scores, mean (SD)						
	Group I		Group II		Group III		P value*
Groups and networks	-0.05	+/-0.15	-0.01	+/-0.14	0.06	+/-0.13	0.855
Trust and solidarity	-0.13	+/-0.15	0.18	+/-0.15	-0.05	+/-0.14	0.266
Collective action and cooperation	0.01	+/-0.14	-0.01	+/-0.25	0.03	+/-0.14	0.994
Information and communication	0.07	+/-0.14	-0.04	+/-0.14	-0.04	+/-0.14	0.818
Social cohesion	-0.09	+/-0.15	0.04	+/-0.14	0.05	+/-0.13	0.756
Empowerment and political action	0.01	+/-0.13	0.02	+/-0.15	-0.03	+/-0.14	0.976
Total score (social capital)	-0.11	+/-0.15	0.07	+/-0.13	0.03	+/-0.14	0.630

## Discussion

In this study, the mean Z-score of the social capital was low in the newly diagnosed PTB patients compared to the HHCs and treatment-completed PTB patients. Similarly, the higher proportion of newly diagnosed PTB patients was in the poorest quartile in social capital. Even though these were not statistically significant, we can see that the participants with better health status had a higher social capital, similar to previous studies [5,11].

Better social networking reduced hospital visits and expenditures on health [12]. Similarly, in our study, the HHCs and treatment-completed PTB group has a lesser proportion of participants in the poorest quartile. The same can be noted in the Z-scores as well. The improving health in TB patients was influenced by the caregivers and social support as mentioned in another study [13]. This indirectly showed that the treatment-completed PTB patients when used as a support system, the health status would improve for the newly diagnosed PTB patients, as implemented in the National Tuberculosis Elimination programme under the Nikshay Mitra initiative.

The trust and solidarity domain also related to social support that can improve health by a better uptake of services and providing informal support and care to the patients by the caregiver [11]. It was shown that health improves as trust increases [14]. There was a greater level of acceptance of preventive measures, such as vaccination, if the beneficiaries had a good trust in the health system and healthcare services [15,16]. Similarly, in our study, we have noted that a higher proportion of HHCs and treatment-completed PTB patients had higher scores in the trust and solidarity domain compared to the newly diagnosed PTB patients.

The information and communication domain plays an important role in the dissemination of information. Information about health issues and services was higher among newly diagnosed PTB patients. This indirectly shows the credibility of work done by the health professional and field staff, which increases the utilization [5].

A higher proportion of study participants were in the poorest quartile in the collective action and cooperation domain across all the groups. Collective action and cooperation as inputs make a better output and improve social capital. This was a part of the recent update in the national programme where these principles were taken up as community mobilization and people's action [2,17].

Social cohesion was higher among the treatment-completed PTB patients. Health outcomes would be improved through social cohesion by reducing the risk of infections and improving health awareness towards healthy behaviour and practices [18,19]. The geographical factor was a major factor as it increased social ties and maintained connection. Social ties were the determining factors in chronic diseases and infectious diseases. Highly connected individuals would have a greater risk of being in the transmission system of infections [20]. However, in our study, newly diagnosed PTB patients had a negative Z-score in social cohesion, which was a contrast to the results of Zelner et al.'s study, where the incidence of diarrhoeal diseases was relatively low among villagers from a remote area [18].

In our study, empowerment and political action were relatively low among the treatment-completed PTB patients and more among the HHCs who had to take care of the PTB patients. Empowerment would mean being aware of rights and responsibilities [21,22]. HHCs were in the position of sharing the needs and protecting the PTB patients. Furthermore, they could make decisions for themselves and their family.

Studies have reported a positive correlation and suggested that the presence of a support system and coordinated efforts acted against the negative factors [11,23]. Even the prevention of risk factors, such as alcohol or substance use, were also available in the literature [24,25]. The level of social capital affected the treatment outcomes among PTB patients [5]. Better social support from friends and family improved the treatment adherence to anti-tubercular treatments [13]. Interaction with the community can also impact health outcomes. As the trust increases, the health improves [11].

Strengths and limitations

Only a few have studied the relationship between social capital and TB disease. To our knowledge, this is the first of its kind in the South Indian population. TB patients were compared with their HHCs since similar socioeconomic and environmental risk factors would be common in them and the comparison would be robust. 

This study was a part of a major study, and the concept of social capital was studied to explore the relationship with disease status. The generalizability of the study results should be made with care as we recruited a small sample size due to feasibility. The question on social capital might have been difficult for a layperson owing to its subjective nature, and the reliability of the response might have been affected.

## Conclusions

The level of social capital was low among newly diagnosed PTB patients compared to their HHCs and treatment-completed PTB patients. In particular, the domains, i.e., groups and networks, trust and solidarity, collective action and cooperation, and empowerment and political action, were low among the newly diagnosed PTB patients. However, better scores among the HHCs, who were the caregivers of newly diagnosed PTB patients, and the treatment-completed patients, i.e., disease-cured patients, showed a negative association between social capital and TB. Therefore, they can be utilized as a social support system for current diseased patients and improve their health status.
